# Age-related differences in social influence on risk perception depend on the direction of influence

**DOI:** 10.1016/j.adolescence.2017.07.002

**Published:** 2017-10

**Authors:** Lisa J. Knoll, Jovita T. Leung, Lucy Foulkes, Sarah-Jayne Blakemore

**Affiliations:** Institute of Cognitive Neuroscience, University College London, Alexandra House, 17 Queen Square, WC1N 3AR, London, United Kingdom

**Keywords:** Adolescence, Development, Social influence, Social norms, Stereotypes, Risk perception

## Abstract

Adolescents are particularly susceptible to social influence. Here, we investigated the effect of social influence on risk perception in 590 participants aged eight to fifty-nine-years tested in the United Kingdom. Participants rated the riskiness of everyday situations, were then informed about the rating of these situations from a (fictitious) social-influence group consisting of *teenagers* or *adults*, and then re-evaluated the situation. Our first aim was to attempt to replicate our previous finding that young adolescents are influenced more by *teenagers* than by *adults*. Second, we investigated the social-influence effect when the social-influence group's rating was more, or less, risky than the participants' own risk rating. Younger participants were more strongly influenced by *teenagers* than by *adults*, but only when *teenagers* rated a situation as more risky than did participants. This suggests that stereotypical characteristics of the social-influence group – risk-prone teenagers - interact with social influence on risk perception.

## 1 Introduction

Other people's beliefs and actions can have a significant impact on our own behaviour. A large body of work has shown that people change their behaviour in order to fit in with others (Berns et al., 2010, Zaki et al., 2011). It has been suggested that people sometimes correct their behaviour and use others' actions as a guideline because they assume that other people's behaviour is more accurate or correct (informational conformity; Deutsch & Gerard, 1955). People also adjust their behaviour due to social norms or the pursuit of acceptance (normative conformity; Deutsch & Gerard, 1955). The degree of conformity is age-dependent, with children and young adolescents showing a higher susceptibility to social influence than adults (Costanzo and Shaw, 1966, Hoving et al., 1969, Knoll et al., 2015).

Adolescence is a period of life during which we become less family-centred and spend more time with friends (Brown, 1990). The amount of time spent with same-sex peers increases between childhood and adolescence, until mid-adolescence (around age 14), when it appears to peak (Lam, McHale, & Crouter, 2014). What their peers think about them starts to have more influence on adolescents' (13–17 years) evaluation of their social and personal worth as compared with children aged 10–12 years (O'Brien & Bierman, 1988). Peers influence decision-making in adolescence: for example, young and mid-adolescents are more likely to engage in risky behaviour when with their peers than when alone (Dishion and Tipsord, 2011, Gardner and Steinberg, 2005). It has been proposed that this heightened peer influence is partly due to adolescents being hypersensitive to peer rejection (Peake et al., 2013, Sebastian et al., 2010, Somerville, 2013) and possibly also to social approval (Foulkes & Blakemore, 2016). This is proposed to lead adolescents to make decisions in the pursuit of social acceptance and avoidance of social exclusion (Blakemore and Mills, 2013, Knoll et al., 2015).

In a previous study, we investigated age-related changes of social influence on risk perception from late childhood through adulthood in a large group of participants aged between 8 and 59 years (Knoll et al., 2015). Participants were asked to rate the riskiness of everyday situations and were then presented with (fictitious) risk ratings of the same situations from other people, either *teenagers* or *adults*. Participants were then asked to rate the riskiness of the situations again. The results showed that all age groups were influenced by other people's opinions: participants of all ages changed their initial risk ratings in the direction of other people's ratings, but this social-influence effect was highest in late childhood and decreased with age. The results also indicated that, while children and adults were more influenced by the opinions of *adults*, young adolescents (aged 12–14 years) changed their ratings more towards the ratings of *teenagers* than towards the ratings of *adults*. In the current study, we employed the paradigm used in Knoll et al. (2015) and asked a new cohort of 590 participants (aged 8–59 years and divided into five age groups, as in the previous study) to rate the riskiness of everyday situations before and after being shown risk ratings from *adults* or *teenagers*. Our first aim was to investigate whether our previous findings could be replicated in a new sample. Specifically, we predicted that the social-influence effect would decrease with age and that young adolescents would be more influenced by *teenagers* than by *adults* (*peer influence hypothesis*).

In our previous study, we suggested that the social-influence effect was due to young adolescents wanting to be accepted by their peer group, rather than trusting the ratings of *teenagers* more than those of *adults* (Knoll et al., 2015). Other studies have indicated that adolescents' real risk-taking behaviour is affected by their perception of their peers' risk-taking behaviour. For example, a study by D'Amico and McCarthy (2006) demonstrated that perceived peer use of cannabis consumption predicted both onset and extent of cannabis use in young/mid-adolescents (aged 10–15 years). Furthermore, Helms et al. (2014) found that mid-adolescents (aged 16 years) often misperceive the degree of risk-taking behaviour of their peers and this misperception is suggested to predict adolescents' own risk behaviour. It could be that adolescents overestimate the risk-taking behaviour of their peers due to the idea that adolescents are generally more risk-taking than other age groups, which is a common stereotype of ‘typical’ adolescents.

The second aim of the current study was to investigate whether the social-influence effect would depend on the direction of other people's risk ratings. Specifically, we analysed the extent to which participants' risk ratings are affected by whether other people (either *teenagers* or *adults*) rated the situations as less or more risky than did the participants. To this end, we separately analysed situations that were rated as less risky, or more risky, by the participants than by the social-influence groups. We were interested in three hypotheses: (i) the degree of social-influence would decrease with age in both directions i.e. when the provided rating of other people was higher than the participants' rating and when it was lower (*directional social influence hypothesis*); (ii) the directional social-influence effect would be different depending on the social influence group (*teenagers* or *adults*; *directional peer influence hypothesis*); and (iii) this directional peer influence would be different between participant age groups (*age-dependent peer influence hypothesis*). Specifically, we predicted that, because of stereotypes about teenage risk-taking, participants would be more likely to increase their risk ratings of a situation if *teenagers* rate the situation as more risky than they did. This might particularly be the case for young adolescents, who were previously found to be more influenced by teenagers than by adults (Knoll et al., 2015).

## 2 Method

### 2.1 Participants

Participants were visitors to the Science Museum in London, UK, on nine days in May and June 2016. Participants were recruited through information screens around the museum publicising the study, and by researchers inviting visitors to take part. Data from 590 participants (mean age = 22.4 years, *SD* = 11.6, age range = 8–59 years; 316 females, 274 males) were included in the analyses. Data from 24 additional participants were excluded because their responses were incomplete, they were interrupted by other visitors during the task or they volunteered information about being diagnosed with a developmental condition, such as autism or dyslexia. Data from 15 participants were excluded because their age was outside our chosen age range (the same age range as in the previous study by Knoll et al., 2015).

Participants were divided into five age groups, as in the prior study by Knoll et al. (2015): 110 children (48 females, mean age: 9.6 years, age range: 8–11), 63 young adolescents (31 females, mean age: 12.8 years, age range: 12–14), 61 mid-adolescents (40 females, mean age: 16.8 years, age range: 15–18), 193 young adults (111 females, mean age: 21.6 years, age range: 19–25), and 163 adults (86 females, mean age: 37.9 years, age range: 26–59). Informed consent was obtained from participants older than 15 years old and from parents of participants under 16 years old. Participants were not compensated for taking part in the study; the study was advertised as an opportunity to volunteer in a real science experiment. The study was carried out in accordance with UCL Research Ethics Guidelines and approved by the University College London ethics committee.

### 2.2 Risk perception task

We used the risk perception task (Knoll et al., 2015) in which participants are presented with 12 risky scenarios, such as ‘Riding a bike without a helmet’ (see Supplemental Material available online for full list of scenarios). The scenarios were designed to be mildly to moderately risky and, critically, to plausibly result in wide variation in risk perception. Thus, each scenario was selected so that it would not be surprising to participants if it were rated as very risky by some people and not risky at all by others. For each stimulus, participants simultaneously read the scenario on the screen and also listened to it via a set of headphones. The auditory stimuli were spoken by an English female researcher and recorded in a soundproof chamber. After recording, stimuli were digitized (sampling rate = 44.1 kHz; bit depth = 16; monaural) and normalized. Statements were accompanied by an image depicting the situation without providing too much contextual information (e.g. a picture of a bicycle).

**Fig. 1:**
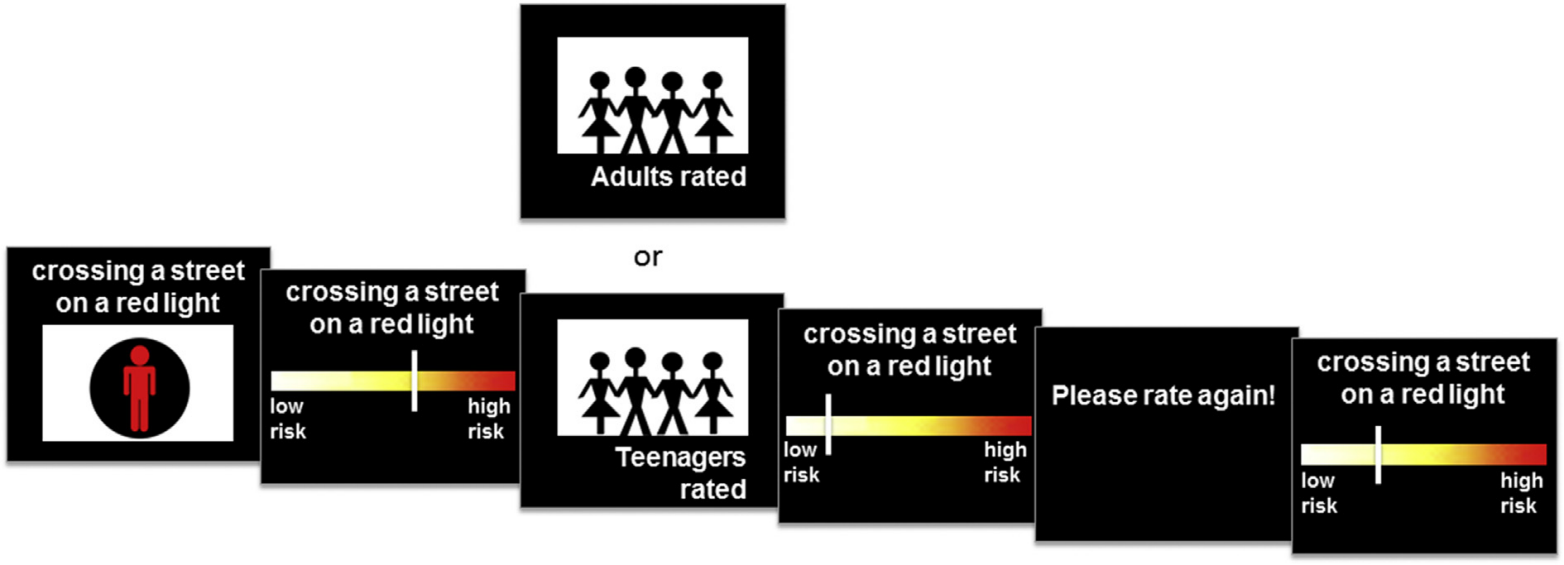
*Illustration of the trial sequence*. Participants were asked to imagine that someone was engaged in an activity (in this example, crossing the street on a red light). They then rated the activity's risk by using a computer mouse to move a slider on a visual analogue scale. After making this rating, participants were shown a risk rating of the same situation that was ostensibly provided by a group of either *adults* or *teenagers* (the social-influence group). The ratings from the social-influence group were actually randomly generated. Finally, participants were asked to rate the same situation again. Adapted and reprinted from (Knoll et al., 2015). (For interpretation of the references to colour in this figure legend, the reader is referred to the web version of this article.)

Participants first read and listened to instructions about the risk perception task. On each subsequent trial, the participant was asked to imagine that someone was engaged in the activity presented. The participant was then asked to rate how risky they thought the scenario was, by using a computer mouse to move a slider to the left side (low risk) or to the right side (high risk) of a colourful visual analogue scale (see Fig. 1). The slider initially appeared at a random position on the scale on each trial to avoid any systematic anchoring bias. There was no time restriction for the first rating: participants had unlimited time to consider their answer and could freely move the slider while they decided, but once they had clicked the mouse the trial moved on. After making the first rating, the participant was shown a risk rating of the same situation by either *adults* or *teenagers* (the social-influence group: *adult* social-influence condition, *teenager* social-influence condition) for 2 s. Participants were told that these ratings were the average answers given by previous study participants. In fact, they were randomly generated and could be lower or higher than the initial rating. This minor deception was approved by the ethics committee.

The factor *direction* was retrospectively determined. In 42% of all trials, the participant's first rating was lower than the provided rating of the social-influence group, and in 58% of all trials it was higher. After being presented with the provided rating from the social-influence group, the participant was asked to rate the same situation again (see Fig. 1). There was no time restriction for the second rating. The subsequent trial started one second after the second rating was provided. A total of 79 different situations were generated for the experiment; 12 (six per social-influence condition) were randomly selected for each participant.

The paradigm employed in the current study was identical to that used in the previous study, with one difference. In the previous version of the task (Knoll et al., 2015), there was a third condition, in which participants saw their own first rating again (rather than a fictitious rating from teenagers or adults) and were asked to rate again. This condition was included previously to check that there was no systematic difference between the age groups in terms of remembering their first rating and to find out the degree to which the participants in different age groups shifted their answers under no social influence. As there were no significant differences between groups in this condition in the previous study, and in order to shorten the task duration, we did not include this control condition here.

The task was programmed using Cogent 2000 (University College London Laboratory of Neurobiology; http://www.vislab.ucl.ac.uk/cogent_2000.php) and run in MATLAB (Version R2012b; MathWorks Inc., Natick MA). Participants were asked to perform the risk perception task after performing a task that assessed face processing, which is not included in this manuscript. The entire set of tasks and instructions took around 12 min. The programme was designed so that all trials required a response, and therefore there were no missing data.

The data were collected in the Live Science area at the back of one of the galleries in the Science Museum, London, UK. This is a secluded and quiet area in which experiments are routinely carried out by research scientists. Three or four researchers were present to oversee testing at all times and there were four laptops available to testing.

### 2.3 Statistical analysis

We employed a 2 × 2 x 5 factorial design with the within-subjects factor social-influence group (*teenagers* vs. *adults*), direction of influence (lower vs. higher ratings) and the between-subjects factor age group (children, young adolescents, mid-adolescents, young adults, adults). All statistical models were estimated in R (R Core Team, 2004), using the lme4 (Bates, Maechler, & Bolker, 2013) and lmerTest (Kuznetsova, Brockhoff, & Christensen, 2013) packages. We ran two linear mixed-effect models to analyse our data, as explained below.

#### 2.3.1 Analysis 1: original study replication

In order to replicate the results of the previous study (Knoll et al., 2015), we first used the same linear mixed-effects model to investigate how much participants changed their risk rating in the direction of others' ratings and whether this social-influence effect is dependent on the source of information (*teenagers* or *adults*) and the age of participants: second rating = first rating + Δrating + Δrating x age group + Δrating x social-influence group + Δrating x social-influence group x age group. As in the original analysis, the current model included the distance between provided rating and first rating (Δrating). Dummy coding was used for age groups. We ran the model twice using the young adults as the baseline group (as in our original model), and with young adolescents as the baseline, to test our hypothesis that young adolescents are more influenced by the social-influence group *teenagers* than *adults*. As these two linear mixed-effects models are sufficient to investigate whether we could replicate the key result of our previous study, it was not necessary to run any additional comparisons. As we were only interested in replicating our original results, we ran the model with these two groups as the baseline groups, and not with any other group as baseline.

#### 2.3.2 Analysis 2: direction factor

We applied a different linear mixed-effects model to investigate the degree to which participants changed their risk ratings in the direction of other people's ratings when the social-influence group rated situations as more or less risky, and the extent to which this change depended on whether the social-influence group consisted of *adults* or *teenagers*. This model incorporated: (a) fixed effects that reflected average effects within and differences between the experimental conditions and (b) random effects that took into account individual variability in the effect of participants' first rating on their second rating and variability between scenarios. The linear mixed-effects model was used to assess the dependence of a participant's second rating on two main predictors: the first rating and the direction of the difference between the rating provided by the social-influence group and the participant's first rating. The difference could be positive (the social-influence group rated the situation as higher risk than the participant) or negative (the social-influence group rated the situation as lower risk than the participant). One trial from one participant was excluded from the analysis, as the difference was zero. The factor *direction* was retrospectively determined. Therefore, we compared likelihood of the full model with the fixed effects *social influence group* by *direction* by *age group* against reduced models with only factor *age group* and a model with factors *age group* and *social influence group*. Model comparisons indicated that the factor social-influence group significantly improved the model (χ2 (5) = 29.67, p < 0.001), but the best model was the full model with all three factors included (χ2 (10) = 1032.2, p < 0.001). To investigate the effect of gender, we applied a model including *gender* (male, female) as an additional factor. Comparing the models, we found that adding this factor improved the model fit (χ2 (20) = 35.578, p = 0.017). However, we decided against a model including *gender* as a factor because adding *gender* to the model would double the number of planned comparisons and we had not planned *a priori,* and do not have sufficient power, to investigate gender differences.

Using the full model with the three-way interaction *age group* by *social influence group* by *direction*, we were particularly interested in: i) whether the previously reported social-influence effect, which was found to decrease with age, would show the same decreasing pattern in trials in which the social-influence group ratings were higher or lower than the participant's first rating (*directional social influence hypothesis*); ii) the directional social-influence effect would be different depending on the social-influence group (*directional peer influence hypothesis*); and iii) this differences would be different between age groups (*age-dependent peer influence hypothesis*). To investigate these questions, the model included interactions between social-influence group, direction of provided rating and age group: second rating = first rating + age group x social influence group x direction. Fixed effects were included for all the main effects and interaction factors in the model. The model did not include an intercept, because an intercept not identical to zero would mean that participants' second rating always increased (or decreased). The effects of the fixed effects on the dependent variable were investigated using an omnibus Type III Wald χ^2^ test.

Planned comparisons were performed to inspect changes in social influence between age groups and social-influence group using the *lsmeans* package (Lenth, 2016). In the first analysis, we investigated age group differences on the degree of social influence irrespective of the factor social-influence group. We analysed situations that were rated as less or more risky by the participants compared to the social-influence groups separately. The *directional social-influence hypothesis* was tested by comparing the degree of influence of the social-influence groups between the five age groups. Each age group's difference was compared with every other age group's difference for both directions, resulting in 20 tests. To investigate our *directional peer-influence hypothesis*, we looked at differences in the degree of influence of the social-influence groups (*adults* vs *teenagers*) within each age group for both higher and lower ratings. The difference for all five age groups was compared for both directions, resulting in 10 tests. The *age-dependent peer-influence hypothesis* was tested by comparing these differences in the degree of influence of the social-influence groups (*adults* vs *teenagers*) effects between the five age groups. Each age group's difference was compared with every other age group's difference for both directions, resulting in 20 tests. All reported results were Bonferroni-corrected for the multiple planned comparisons within each hypothesis.

## 3 Results

### 3.1 Analysis 1: original study replication

We found a significant interaction between age group and Δrating (difference between provided rating and first rating), indicating that participants changed their risk ratings in the direction of the provided ratings and this effect differed between age groups, replicating previous findings (Knoll et al., 2015). There were age differences between children and young adolescents (t(895) = 2.63, *p* = 0.009) and between young adolescents and mid-adolescents (t(785) = −3.70, *p* < 0.001) and no age differences between mid-adolescents and young adults (t(737) = 1.73, *p* = 0.08), or between young adults and adults (t(717) = −1.30, *p* = 0.2). This partially replicates the previous findings, which found significant decreases in social influence between each successive pair of age groups (for table of model summaries see Supplementary Tables 1 and 2).

**Fig. 2:**
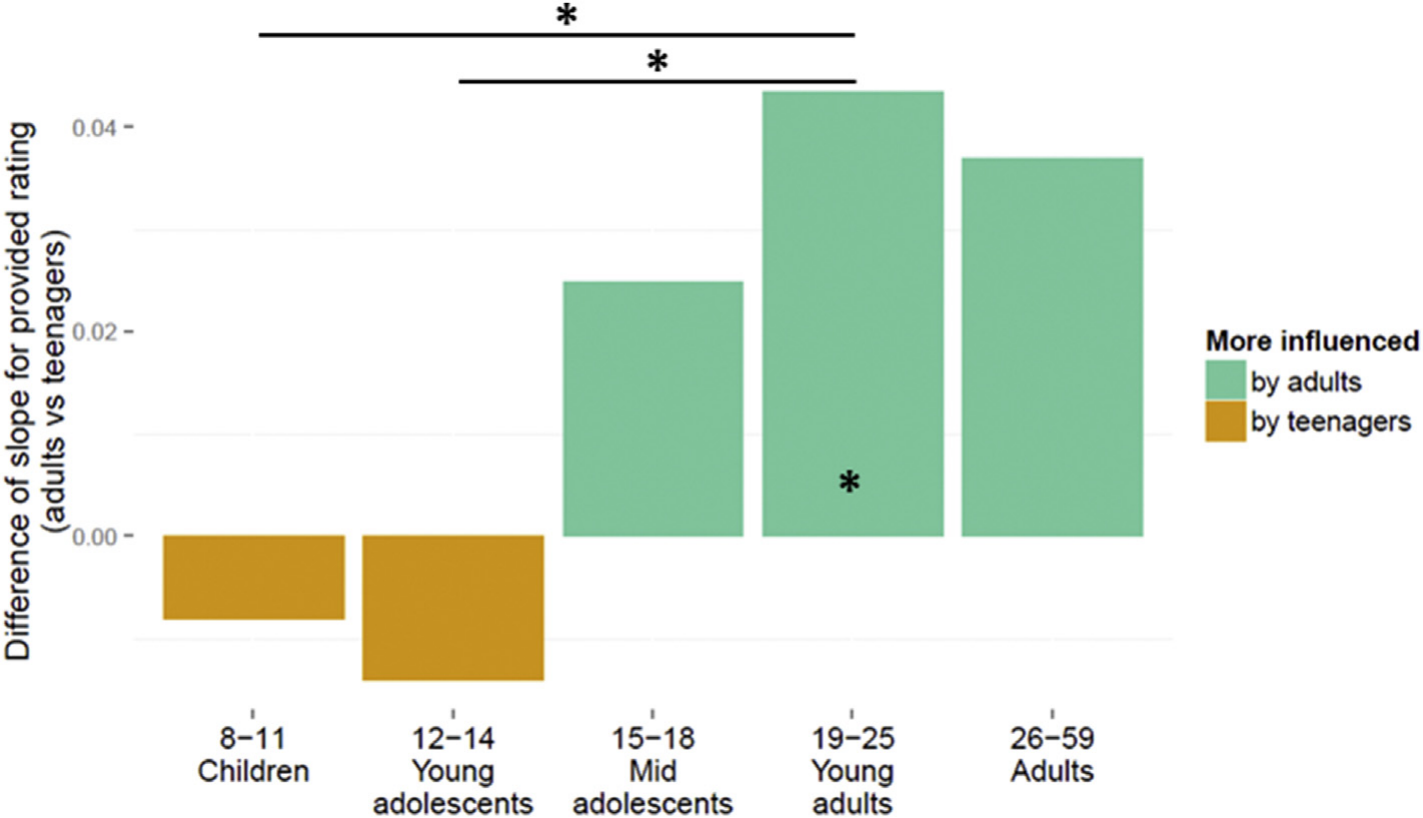
The graph presents the difference between the two slopes for the average change in risk rating after seeing the ratings of the social-influence groups, predicted by using the estimates of the linear mixed-effect model, for five age groups. A positive difference indicates a greater degree of influence by the social-influence group *adults* than by *teenagers*. A negative difference indicates a greater degree of influence by the social-influence *teenagers* than by *adults*. Asterisks indicate significant main effect of peer-influence (*adults* vs *teenagers*) within age group and interaction on peer-influence effects between age groups (* p < 0.05).

Young adults were more influenced by the opinion of *adults* than *teenagers* (in young adults t(6494) = 3.59, *p* < 0.001), replicating the previous results. In the previous study, 12–14 year olds were more influenced by *teenagers* than by *adults* and this was not the case for any other age group. In the current study, although the social-influence group effect in 12–14 year olds appears to be in the same direction as the previous findings (Fig. 2 shows that overall, young adolescents were more influenced by *teenagers* than by *adults*), the direct comparison between social-influence group *teenagers* and *adults* was not significant for this age group (t(6499) = −0.65, *p* = 0.5). However, the interaction between age group (two levels: young adults versus young adolescents) and social-influence group (two levels: *teenagers* vs *adults*) was significant (t(6461) = −2.32, *p* = 0.020), as was the interaction between age group (young adults vs children) and social-influence group (two levels: *teenagers* vs *adults*; t(6537) = −2.55, *p* = 0.011). This shows that, whereas adults were more influenced by *adults* than by *teenagers*, the effect was reversed in the young adolescent age group, replicating our previous findings.

### 3.2 Analysis 2: direction factor

#### 3.2.1 Directional social-influence hypothesis

**Fig. 3:**
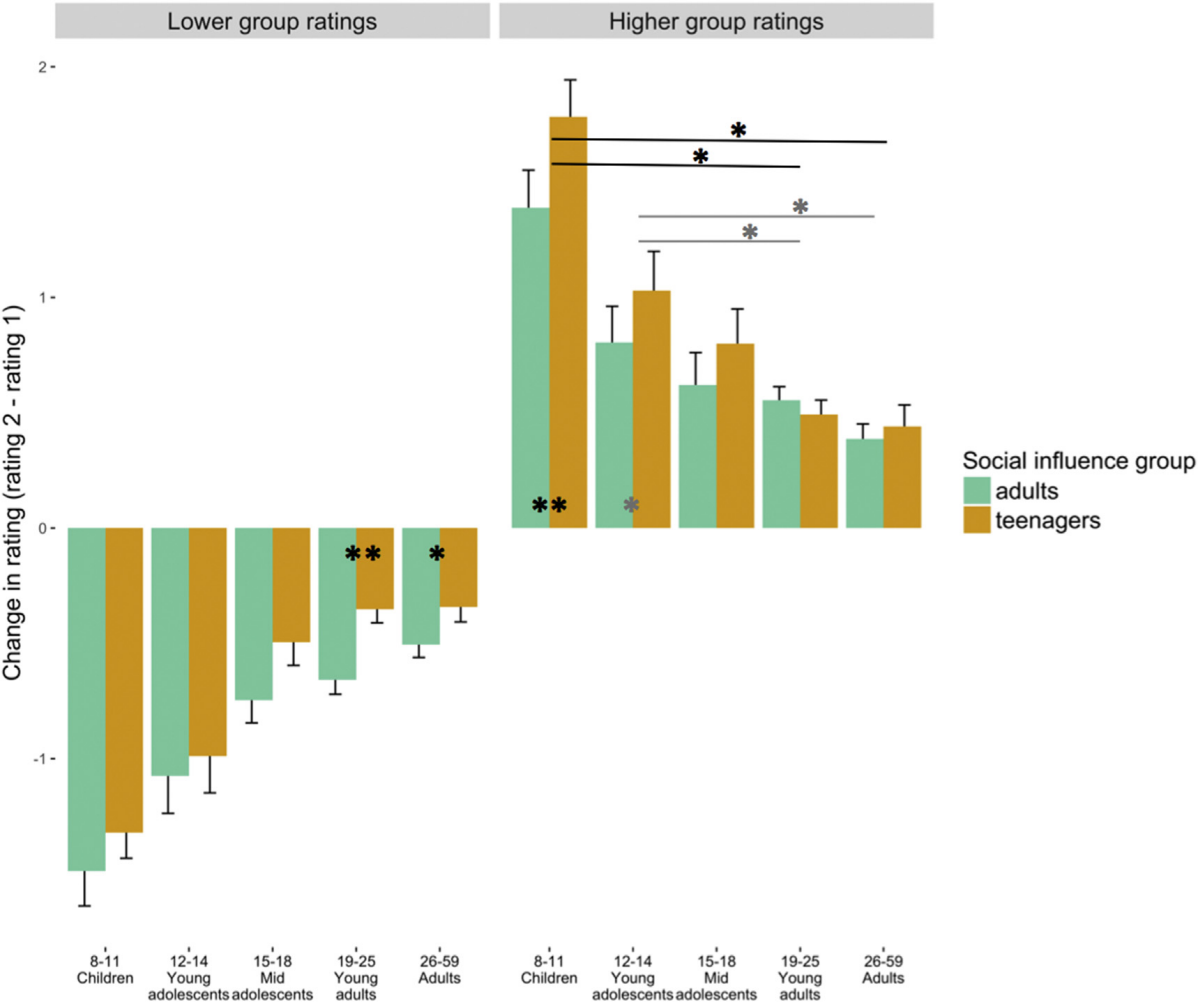
The graph shows the average differences in rating (ratings 2 minus rating 1) with standard error bars. Results are shown separately for the *adult* social-influence condition and the *teenager* social-influence condition, for each age group. Bars on the left (‘Lower group ratings’) were from the conditions in which the social-influence group rated risk lower than participants' initial rating. Bars on the right (‘Higher group ratings’) were from the conditions in which the social-influence group rated risk higher than participants' initial rating. Asterisks indicate significant main effect of peer-influence (*adults* vs *teenagers*) within age group and interaction on peer-influence effects between age groups (** p < 0.01, * p < 0.05, black: Bonferroni corrected result; grey: uncorrected result).

We used a linear mixed-effects model that incorporated the direction of the social-influence group's ratings as a factor to investigate how much participants change their rating if the social-influence group rated a situation as lower risk or higher risk than did the participants. We observed a significant main effect of age (*χ*^2^(5) = 52.89, *p* < 0.001) and planned comparisons revealed that social influence decreased from childhood to adulthood both when the provided rating was higher than the participant's rating and when it was lower (see Fig. 3). However, not all comparisons survived alpha correction for the 20 multiple comparisons conducted to test this hypothesis (see Table 1). First, when the social-influence group rated situations as less risky: children were more influenced than young adolescents (z = −3.52, *p* < 0.001), mid-adolescents (z = −5.83, *p* < 0.001), young adults (z = −10.38, *p* < 0.001) and adults (z = −10.80, *p* < 0.001); and young adolescents were more influenced than young adults (z = −4.83, *p* < 0.001) and adults (z = −5.30, *p* < 0.001). Second, when the social-influence group rated situations as more risky: children were more influenced than young adolescents (z = 4.60, *p* < 0.001), mid-adolescents (z = 7.07, *p* < 0.001), young adults (z = 10.86, *p* < 0.001) and adults (z = 11.30, *p* < 0.001); and young adolescents were more influenced than young adults (z = 3.87, *p* < 0.001) and adults (z = 4.52, *p* < 0.001). While we did not find a continuous decrease throughout all five age groups, we found that children were more influenced than young adolescents and both age groups were significantly more influenced by other people's risk ratings compared to young adults and adults, in both directions.

**Table 1:** Planned contrasts showing age differences of social-influence effect. Table shows estimates, standard error (SE), z values, p values and p values Bonferroni-corrected for 20 comparisons, for all age group comparisons.

	Estimate	SE	z value	*p*	*p* (bonf.)
lower: children vs. young adol	−0.30	0.08	−3.52	<0.001	<0.001
lower: children vs. mid-adol	−0.50	0.09	−5.83	<0.001	<0.001
lower: children vs. young adults	−0.68	0.07	−10.38	<0.001	<0.001
lower: children vs. adults	−0.72	0.07	−10.80	<0.001	<0.001
lower: young adol. vs. mid-adol	−0.20	0.10	−2.06	0.039	0.780
lower: young adol. vs. young adults	−0.38	0.08	−4.83	<0.001	<0.001
lower: young adol. vs. adults	−0.42	0.08	−5.31	<0.001	<0.001
lower: mid-adol. vs. young adults	−0.18	0.08	−2.32	0.020	0.400
lower: mid-adol. vs. adults	−0.22	0.08	−2.84	0.005	0.100
lower: young adults vs. adults	−0.04	0.06	−0.78	0.437	>1
higher: children vs. young adol	0.48	0.10	4.62	<0.001	<0.001
higher: children vs. mid-adol	0.73	0.10	7.09	<0.001	<0.001
higher: children vs. young adults	0.85	0.08	10.90	<0.001	<0.001
higher: children vs. adults	0.93	0.08	11.33	<0.001	<0.001
higher: young adol. vs. mid-adol	0.24	0.12	2.11	0.035	0.700
higher: young adol. vs. young adults	0.37	0.09	3.88	0.000	<0.001
higher: young adol. vs. adults	0.44	0.10	4.54	<0.001	<0.001
higher: mid-adol. vs. young adults	0.12	0.09	1.34	0.181	>1
higher: mid-adol. vs. adults	0.20	0.10	2.09	0.037	0.740
higher: young adults vs. adults	0.08	0.07	1.11	0.267	>1

#### 3.2.2 Directional peer-influence hypothesis

The two-way interaction between age group and social-influence group was not significant (*χ*^2^(4) = 6.25, *p* = 0.18). However, planned comparisons revealed that young adults and adults were more influenced by the social-influence group *adults* compared to *teenagers* when the provided risk ratings were lower than their own ratings. In contrast, children and young adolescents were more influenced by the social-influence group *teenagers* than *adults* when the provided risk ratings were higher than their own ratings. Only the effect in children (z = −3.79 *p* = 0.001), young adults (z = −4.01, *p* = 0.001) and adults (z = −2.87,*p* = 0.04) survived Bonferroni correction for the 10 comparisons conducted to test this hypothesis (for planned comparisons see Table 2).

**Table 2:** Planned contrasts showing differences of peer influence effect within age group. Table shows estimates, standard error (SE), z values, p values and p values Bonferroni-corrected for 10 comparisons, for all age group comparisons.

	Estimate	SE	z value	*p*	*p* (bonf.)
lower: SI group: children	−0.12	0.09	−1.31	0.191	>1
lower: SI group: young adol.	−0.14	0.12	−1.18	0.238	>1
lower: SI group: mid-adol.	−0.22	0.13	−1.74	0.082	>1
lower: SI group: young adults	−0.30	0.07	−4.01	<0.001	0.001
lower: SI group: adults	−0.22	0.08	−2.87	0.004	0.040
higher: SI group: children	−0.46	0.12	−3.79	<0.001	0.001
higher: SI group: young adol.	−0.35	0.16	−2.25	0.025	0.250
higher: SI group: mid-adol.	−0.18	0.15	−1.21	0.228	>1
higher: SI group: young adults	0.06	0.08	0.69	0.493	>1
higher: SI group: adults	0.08	0.09	0.81	0.415	>1

#### 3.2.3 Age-dependent peer-influence hypothesis

The three-way interaction between age group, direction and social-influence group was significant (*χ*^2^(4) = 18.88, *p* < 0.001), indicating that the different social-influence groups, *teenagers* or *adults*, and the direction of the provided rating, had a significant impact on the second rating, and this effect differed between age groups. Planned comparisons investigating the interactions between social-influence groups and age groups revealed that younger age groups were significantly more influenced by *teenagers* than older age groups. Significant interactions were found between children and young adults, between children and adults, between young adolescents and young adults as well as between young adolescents and adults, when the social-influence group rated situations as more risky than did participants. That is, younger age groups were significantly more influenced by teenagers than by adults in more risky conditions. Only planned comparisons in children vs young adults (z = 3.51, *p* = 0.008), and children vs adults (z = 3.48, *p* = 0.008), survived Bonferroni correction for the 20 comparisons conducted to test this hypothesis (for planned comparisons see Table 3).

**Table 3:** Planned contrasts showing differences of peer influence effect between age groups for lower and higher ratings, respectively. Table shows estimates, standard error (SE), z values, p values and p values Bonferroni-corrected for 20 comparisons, for all age group comparisons.

	Estimate	SE	z value	*p*	*p* (bonf.)
lower: SI group: children vs. young adol	−0.02	0.15	−0.16	0.871	>1
lower: SI group: children vs. mid-adol	−0.11	0.16	−0.68	0.500	>1
lower: SI group: children vs. young adults	−0.18	0.12	−1.54	0.124	>1
lower: SI group: children vs. adults	−0.11	0.12	−0.88	0.377	>1
lower: SI group: young adol. vs. mid-adol	−0.08	0.18	−0.46	0.644	>1
lower: SI group: young adol. vs. young adults	−0.16	0.14	−1.10	0.273	>1
lower: SI group: young adol. vs. adults	−0.08	0.14	−0.56	0.574	>1
lower: SI group: mid-adol. vs. young adults	−0.07	0.15	−0.50	0.621	>1
lower: SI group: mid-adol. vs. adults	0.00	0.15	0.01	995	>1
lower: SI group: young adults vs. adults	0.07	0.11	0.70	0.478	>1
higher: SI group: children vs. young adol	0.10	0.20	0.52	0.606	>1
higher: SI group: children vs. mid-adol	0.28	0.19	1.44	0.150	>1
higher: SI group: children vs. young adults	0.51	0.15	3.51	<0.001	0.020
higher: SI group: children vs. adults	0.53	0.15	3.48	<0.001	0.020
higher: SI group: young adol. vs. mid-adol	0.17	0.22	0.80	0.422	>1
higher: SI group: young adol. vs. young adults	0.41	0.18	2.30	0.021	0.420
higher: SI group: young adol. vs. adults	0.43	0.18	2.35	0.019	0.380
higher: SI group: mid-adol. vs. young adults	0.24	0.17	1.39	0.166	>1
higher: SI group: mid-adol. vs. adults	0.26	0.18	1.45	0.146	>1
higher: SI group: young adults vs. adults	0.02	0.12	0.17	0.869	>1

## 4 Discussion

The current study investigated the social-influence effect on risk perception in adolescence. We were particularly interested in examining age-related differences in social-influence effects as a function of social influence group (*teenagers* or *adults*) as well as the direction of influence (more or less risky).

Our first aim was to replicate the results of our previous study (Knoll et al., 2015) in a new sample of 590 participants, with a similar age and gender distribution. In the previous study, 55.6% of participants were female and the mean age was 23.4 years, whereas in the current study, 53.6% of participants were female and the mean age was 22.4 years. The current study partially replicated the previous findings in three ways. First, as in the previous study, the current study found that risk perception was influenced by the risk ratings of other people. Second, the social-influence effect decreased from late childhood to late adolescence. It has been suggested that participants adjust behaviour due to informational conformity, that is the pursuit of accuracy (Deutsch & Gerard, 1955). Participants might have taken into account risk perception of other people and adapted their ratings accordingly. As the degree of conformity was found to be dependent on age (Engelmann et al., 2012, Hoving et al., 1969), it is possible that older participants are more confident in their risk ratings as they have experienced these kinds of situation more often than younger age groups. In contrast to the previous study, we did not observe further significant decrease of social influence into adulthood. Thus, while our current results provide further evidence that social influence decreases from childhood to mid-adolescence, our previous results showing a decrease into adulthood should be interpreted with caution.

Third, there was an interaction between age group and social-influence group such that young adults were more influenced by *adults* than by *teenagers,* whereas this peer-effect was reversed in young adolescents. Note that, in contrast to the previous study, this reversal was also present in children. In the previous study, we suggested that children might be more influenced by the social-influence group *adults* than by *teenagers* because adults represent authority figures, who have experienced the situations more often than teenagers. It could be that the group of children tested in this second study happened to be more frequently surrounded by teenagers, for example, they may have had more older siblings than those in the first study. Of course, this is only speculation and further studies would need to be conducted to investigate this idea.

Previous research has shown that, when adolescents are with peers they are more likely to take risks, such as engaging in reckless behaviour and experimenting with drugs, alcohol and cigarettes, compared to when they are alone (Reniers et al., 2017). Lab experiments have shown that adolescents are more likely to take driving risks when with friends, compared with when alone; in contrast, adults' (24 years and over) driving risks are unaffected by peers (Gardner & Steinberg, 2005). This is mirrored by findings from data from car accidents, which indicate that the risk of accidents for young drivers is heightened when they have a passenger in the car (Chen, Baker, Braver, & Li, 2000). Studies in Hong Kong, for example, have shown that having friends who smoke or drink alcohol is the biggest predictor of adolescent smoking and drinking (Loke & Mak, 2013). A longitudinal study involving adolescents (aged 10–15 years at the start of the study) in California, showed that *perceived* peer cannabis use predicts onset and extent of the adolescent's own cannabis use over the next three years; a similar relationship was found for alcohol use (D'Amico & McCarthy, 2006). Many previous studies do not include a large age range and therefore cannot draw conclusions about age differences in social influence. Our findings extend this previous research by including a large age range and showing that young adolescents' risk perception is particularly influenced by the risk ratings of *teenagers* (Fig. 2).

The second aim of the current study was to understand whether social influence is affected by the *direction* of other people's risk ratings and whether this differs as a function of the age of participants. This new analysis showed that risk perception was influenced by lower and higher risk ratings of the social-influence group and such influence decreased from childhood to adulthood and was in line with our *directional social influence hypothesis*. In addition, we found that the directional peer-influence effect was dependent on whether the social-influence group consisted of *teenagers* or *adults* (*directional peer influence hypothesis*). Specifically, children were more influenced by *teenagers* when *teenagers* rated the situation as more risky than the participants did. The same trend was found in young adolescents, who were more influenced by *teenagers* when *teenagers* rated the situation as more risky than the participants' ratings (although note that planned comparisons in young adolescents did not survive Bonferroni correction). In contrast, young adults and adults were more influenced by *adults* than by *teenagers* when the social-influence group *adults* rated the situation as less risky than the participants (*age-dependent peer influence hypothesis*).

One explanation for this opposite pattern is that it is due to shared beliefs about members of a particular group, which in this study were the social-influence groups *teenagers* and *adults*. Shared beliefs can be stereotypes, which are socially shared characteristics and expectations and are used to predict behaviour of members of a group (Stangor and Lange, 1994, Yzerbyt, 2016). This study was framed to participants as a study about risk-taking and it is possible that stereotypes about teenage behaviour, such as the notion that teenagers are prone to risk-taking (Buchanan & Holmbeck, 1998), drove the directional peer-influence effect. In this case, children and young adolescents were more influenced by *teenagers* than by *adults* when stereotyped ‘risk-prone teenagers’ rated situations as more risky than the participants themselves rated the situation. In other words, we are suggesting that children and young adolescents pay more attention when *teenagers* consider a situation as high risk as, due to stereotypes about teenage risk-taking, it might be surprising to them that *teenagers* rate a situation as very risky. Specifically, if an adolescent learns that his or her peers consider a situation very risky, he or she might pay heightened attention to that information because it might be especially surprising that other teenagers – a group they stereotypically expect to take risks – rate a situation as very risky. This surprising information might make the adolescent pay attention to this information and to adjust their own ratings accordingly. This interpretation is supported by a behavioural study that indicated that social influence could be driven by perception of social norms (Helms et al., 2014). Helms and colleagues found that mid-adolescents (aged 16 years) tend to overestimate the risk-taking behaviour of their peers, especially high status peers. As Helms and colleagues did not include young adolescents in their study, we do not know whether the same effect would be found in young adolescents. Similarly, older age groups in the current study were more likely to follow adult advice when the social-influence group adults rated situation as less risky. This might be due to the social-influence group *adults* being considered more experienced and trustworthy or because older age groups expected *teenagers* to underestimate risk.

### 4.1 Limitations

There were a number of limitations to our study. First, due to the restrictions imposed by the Science Museum, where testing was carried out, we were unable to collect information about participant characteristics such as socio-economic status and ethnicity. Based on information provided by the Science Museum, 3.22 million people visited the Museum in 2016/2017 and, of these, 58% visited as part of a family and 42% independent adults. Approximately 59% of independent adult visitors were tourists, so our sample is not likely to be representative of the British population in terms of culture or ethnicity. As cultural differences could have an impact on social influence, this should be explored in future studies. Second, we do not have information about the visitors who volunteered for our study compared with those who did not volunteer. Visitors were informed about the study via information screens throughout the museum and there was no way of knowing how many visitors saw the information screens, or were invited to take part but declined. It is possible that visitors who volunteered were a self-selecting group, and might differ from the average Science Museum visitor, perhaps in terms of motivation and language. Third, due to time constraints, we did not specifically ask about developmental conditions, such as dyslexia and autism spectrum conditions, and instead we excluded data from participants who volunteered this information. It is possible that individuals with developmental conditions were included in our sample, and this might have affected the data. Fourth, although the testing area was relatively quiet, calm and secluded, other visitors occasionally pass by and it is possible to hear noises from the nearby exhibits. We asked parents and other visitors to keep their distance from participants while they were taking part, and participants were also asked to wear noise-cancelling headphone to avoid distractions.

### 4.2 Implications

Understanding the effect of peer influence on risk-taking might help to improve public health interventions and specifically target younger age groups. Public health advertising aimed at young people's predilection for risky behaviours tends to focus on the health risks of these behaviours, but focusing on social norms and peer expectations might have more impact on adolescent behaviour. This was supported by a recent public health study that looked at the influence of social norms on bullying behaviour and conflict in schools (Paluck, Shepherd, & Aronow, 2016). Fifty-six middle schools (with children aged 11–16 years) in the state of New Jersey, USA, were included, with half of the schools being assigned at random to an anti-bullying programme. In this programme, a number of students in each year participated in an anti-conflict programme, which involved a researcher working with the students to understand the negative effects of bullying. The students on the programme were encouraged to lead grassroots anti-bullying campaigns in their schools and become the public face of opposition to bullying. Compared with control schools, in which no special anti-bullying programmes had been introduced, reports of student conflict at the schools that had received the student-led anti-bullying programme were reduced by 30%. Furthermore, when the anti-bullying campaign was led by more popular students, it had a greater positive effect on behaviour. The study reveals the power of peer influence in changing social norms of acceptable behaviour.

## 5 Conclusion

The results of the current study suggest that stereotypical characteristics of the social-influence group interact with social-influence on risk perception. The findings indicate that children and young adolescents place different values on the opinions of the social-influence groups than do older people, and attach more importance to heightened risk perception of *teenagers*. We propose that socially shared expectations of specific group members, in this study the social-influence group *teenagers*, affect the degree of social influence on risk perception. Future research should explore other key factors that drive and affect social influence and how these factors are valued across development.

## Author contribution statement

L.J.K and S.-J.B. designed the study. L.J.K., J.T.L. and L.F. organized testing and collected the data. L.J.K. analysed the data. L.J.K, J.T.L., L.F and S.-J.B. wrote the manuscript.
