# Supplementary material for: Age-related differences in social influence on risk perception depend on the direction of influence
Source: J Adolesc. 2017 Oct;60:53–63. doi: 10.1016/j.adolescence.2017.07.002 (PMC5614112; doi:10.1016/j.adolescence.2017.07.002)
Supplement: Supplementary file 1 [file mmc1.docx]

**Table S1**

**Summary linear mixed-effects model with young adults as baseline. (A.) Slopes and test results**. Table shows fixed effects are summarized showing estimates, standard error (SE), df, t-values and p-values for the model fixed predictors and their interactions. The random effects summary table shows group, name of the variable, variance, and standard deviation (SD). Abbreviation: first rating (r1); Δrating (Δr); age group (age); social influence group (social influence).

| **Slopes and tests of the individual fixed effects** |  |  |  |  |  |  |
| --- | --- | --- | --- | --- | --- | --- |
| **Fixed effects** | **Estimate** | **SE** | **df** | **t** | **p** |  |
| r1 | 0.98 | 0.01 | 732 | 115.86 | <.001 | *** |
| Δr | 0.12 | 0.01 | 745 | 8.83 | <.001 | *** |
| Δr:children | 0.25 | 0.03 | 653 | 10.78 | <.001 | *** |
| Δr:young adol | 0.17 | 0.03 | 1031 | 6.71 | <.001 | *** |
| Δr:mid-adol | 0.05 | 0.03 | 737 | 1.83 | .08 |  |
| Δr:adults | -0.03 | 0.02 | 717 | -1.30 | .20 |  |
| Δr:social influence | 0.02 | 0.01 | 6494 | 3.59 | <.001 | *** |
| Δr:children:social influence | -0.02 | 0.01 | 6537 | -2.55 | .01 | * |
| Δr:young adol:social influence | -0.03 | 0.01 | 6461 | -2.32 | .02 | * |
| Δr:mid-adol:social influence | -0.01 | 0.01 | 6521 | -0.72 | .47 |  |
| Δr:adults:social influence | -0.01 | 0.01 | 6500 | -0.36 | .72 |  |
|  |  |  |  |  |  |  |
| **Random effects** |  |  |  | **Variance** | **SD** |  |
| Δr |  |  |  | 0.029 | 0.171 |  |
| r1 |  |  |  | 0.015 | 0.123 |  |
| residual |  |  |  | 1.294 | 1.138 |  |
